# Managing Risk of Non-Communicable Diseases in Women with Bulimia Nervosa or Binge Eating Disorders: A Randomized Trial with 12 Months Follow-Up

**DOI:** 10.3390/nu10121887

**Published:** 2018-12-03

**Authors:** Therese Fostervold Mathisen, Jorunn Sundgot-Borgen, Jan H. Rosenvinge, Solfrid Bratland-Sanda

**Affiliations:** 1 Department of Sports Medicine, Norwegian School of Sport Sciences; Sognsvegen 220, 0806 Oslo, Norway; jorunn.sundgot-borgen@nih.no; 2 Department of Psychology, Faculty of Health Sciences, UiT- the Arctic University of Norway, N-9037 Tromsø, Norway; jan.rosenvinge@uit.no; 3 Department of Sports and Physical Education and Outdoor Sciences, University of South-Eastern Norway, N-3800 Bø in Telemark, Norway; solfrid.bratland-sanda@usn.no

**Keywords:** physical fitness, visceral adipose tissue, obesity, eating disorders, nutrition, physical activity, exercise, bulimia, binge eating disorder

## Abstract

Persons with bulimia nervosa (BN) or binge eating disorder (BED) have an elevated risk of non-communicable diseases (NCDs). However, lowering this risk is rarely addressed in standard cognitive-behavioral treatment (CBT). We aimed to compare CBT with an intervention combining physical exercise and dietary therapy (PED-t), and hypothesized that the PED-t would do better than CBT in lowering the risk of NCD both initially and longitudinally. In this study, 164 women with bulimia nervosa or binge eating disorder were randomly assigned to 16-weeks of outpatient group therapy with either PED-t or CBT. Body composition (BC) was measured by dual-energy X-ray absorptiometry. Measures of physical fitness (VO_2_peak and one repetition maximum (1RM) in squats, bench press, and seated row) were also recorded. All measurements were completed baseline, post-treatment, and at 6- and 12-month follow-ups, respectively. Our results showed that PED-t improved more than CBT on mean (99% CI) absolute Vo2peak; 57,2 (84.4, 198.8) mL (*g* = 0.22, *p* < 0.001) post-treatment. There were small to medium long-term differences in 1RM after PED-t compared to CBT. BC deteriorated in both groups during follow-up. Neither the PED-t nor the CBT lowered the risk for NCDs. Clearly, other approaches need to be considered to promote physical fitness and lower the risk of NCDs among individuals with BN and BED.

## 1. Introduction

Poor physical fitness is a major public health challenge, and it has been found to be prevalent among those suffering from bulimia nervosa (BN) or binge eating disorders (BED) [1,2]. Poor physical fitness may in return increase the risk of non-communicable diseases (NCDs) such as type 2 diabetes, obesity, and cardiovascular symptoms [3]: risk profiles previously identified in BN and BED [4,5,6,7,8,9]. 

It is well documented that lifestyle interventions focusing on diet and physical activity may reduce the risk of obesity and many NCDs [10]. However, in the treatment of BN, and notably, anorexia nervosa, there is a reluctance to focus on physical activity because of fear of triggering or exacerbating dieting, compulsive exercise, and other eating disorder symptoms (ED-symptoms) [11,12]. Further, with respect to BN, and BED in particular, a need to focus on physical activity due to a risk of obesity is hampered by the fact that many treatment guidelines recommend postponing such a focus until eating disorder symptoms have been successfully treated [12,13]. Another problem regarding the limited attention to somatic comorbidity during treatment of BN and BED is the poor specificity of body mass index (BMI) categories and the waist-circumference ratio to detect overweight problems. Thus, “masked obesity” in terms of a morbid body composition may occur in cases of a high body fat percentage [2,14] and a high level of visceral adipose tissue despite normal BMI [2]. For these reasons, physical fitness is understudied and poorly addressed in the treatment of BN and BED. As a consequence, many treatment guidelines related to physical health are somewhat skewed towards the management and treatment of severe anorexia nervosa [12]. 

Cognitive-behavioral therapy (CBT) is recognized as a gold standard treatment for eating disorders due to its capacity to reduce concern for body weight and shape, to improve self-esteem, self-efficacy, and self-compassion, and to affect regulation [15,16]. Such cognitive-affective changes lead to behavioral changes like reduced bingeing and increased health-related physical activity [17,18]. However, in a previous clinical study on eating disorders, we showed that a CBT-based treatment approach, in fact, did not produce changes in physical fitness and body composition [19]. A possible explanation of such findings is that CBT for eating disorders lacks a specific focus on weight loss and physical fitness and how to improve it [20]. On the other hand, several studies as well as a systematic review have reported that including physical activity in treatment may reduce BN and BED symptoms [21,22,23,24,25,26,27,28]. However, these studies have not reported data on changes in physical fitness, body composition, and other variables related to NCDs. In sum then, with a lack of focus on addressing physical fitness in the CBT manuals, one may hypothesize that a CBT treatment for BN and BED may not lower the risk of NCDs. Consequently, it is reasonable to hypothesize that a treatment of BN and BED that has an explicit focus on physical activity and that has been found to reduce compulsive exercise [27] and to match CBT in overall treatment efficiency [28] may be more effective than a CBT treatment in terms of lowering the risk of NCDs. 

In addition to investigating the effect of a new treatment program for BN and BED on ED-symptoms, i.e., bingeing/purging, and an overvaluation on the need to control food intake, body weight and shape (previously reported) [27,28], we also wanted to investigate the acute and long-term effects of physical exercise and dietary therapy (PED-t) or CBT on physical fitness, body composition (BC), and risk of NCDs. We hypothesized that PED-t improves physical fitness and BC more than CBT, hence resulting in a more favorable risk profile for NCDs.

## 2. Materials and Methods 

### 2.1. Subjects

We recruited females with BN or a BED to a 16-week outpatient group treatment, arranged at the Norwegian School of Sport Sciences (NSSS) during 2014–2016 [28]. The recruitment was directed through general practitioners, national and regional media, and social media. 

All interested were interviewed by telephone using the Eating Disorder Examination questionnaire (EDE-q) [29] and the mini international neuropsychiatric interview (MINI) [30]. To optimize group heterogeneity, only women aged 18–40 years with BN or a BED were eligible. Participation in the study was restricted to women who were not currently pregnant and who had a BMI to 17.5–35 in order to reduce the risk of injuries and other complications if being randomized to the PED-t, as women who do not meet these criteria may not be able to follow a standardized exercise and dietary treatment protocol or to follow group exercise with minor room for individual adjustments. The study also excluded competitive athletes, those with comorbid mental disorders in need of other treatments, or those living distances to the study site that prevented regular attendance. 

**Figure 1:**
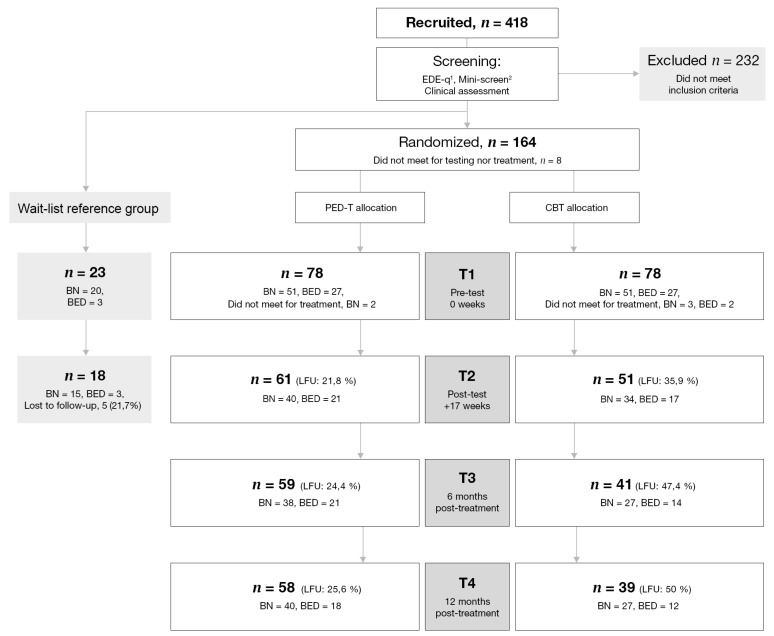
Recruitment, retention, and attrition during test periods. Overview of recruitment, screening, randomization, and attendance to the baseline (T1), post-treatment (T2), and follow-up measures (T3–T4). PED-t: physical exercise and dietary therapy; CBT: cognitive behavior therapy; BN: bulimia nervosa; BED: binge-eating disorder; EDE-q: eating disorder examination questionnaire; LFU: lost to follow up; ^1^ Fairburn and Beglin, 2008 [29]; ^2^ Sheehan, Lecrubier, Sheehan, et al., 1998 [30].

As previously reported [31], power analysis indicted a need for a minimum of 144 (72 per group) participants. We randomized 164 of those 419 who initially responded to recruitment to either PED-t (*n* = 76) or CBT (*n* = 73) (Figure 1). A randomization list with block size of eight was produced by www.randomizer.org by a statistician not connected to the study, and a fellow worker with no involvement in the trial randomized participants to either CBT or PED-t. All participants were informed about the outcome of the randomization after they had completed all baseline measures.

### 2.2. Treatments 

The 16-week CBT and PED-t group-based therapies consisted of 20 sessions, each of 90 min duration, with five to eight participants in each group. A detailed account of the therapies and design are described elsewhere [31].

The manual-based CBT rests on a transdiagnostic model positing generic characteristics across ED-diagnoses [32]. Psychologists certified in CBT, with at least 10 years of experience, were conducting this therapy. The therapists were supervised by one senior therapist (PhD, psychologist and scientist) with more than 20 years of experience in CBT.

The PED-t consisted of a combination of dietary therapy and progressive resistance training (RET) and high intensity interval running (HIIT) [31]. The dietary manual focused on education on nutrition, group discussions, and practical skills in overcoming challenges in establishing regular daily eating patterns. We designed the whole-body, progressive resistance exercise program with two sessions per week arranged in three modules across the 16 weeks following basic principles for increases in maximal muscle strength (Table 1) [33]. The HIIT was designed with session intensity according to a pyramid structure, with progressive exercise load throughout the 16 weeks, following general recommendation for cardio respiratory fitness (CRF) improvement [34]. 

Specialists in physical exercise and in dietetics (MSc), all of whom were instructed by one experienced therapist (dietitian and exercise physiologist), were responsible for this therapy. 

Participants were requested to complete training diaries throughout the treatment period in order to analyze adherence rate to training intervention.

### 2.3. Outcomes and Procedures 

We measured body composition, physical activity, cardiorespiratory fitness (CRF), and maximal muscle strength during baseline and all follow-up test periods. Before all measure points, participants had to fast and to use passive transportation to the laboratories at the NSSS from 7:30 to 10:00 AM.

#### 2.3.1. Body Composition 

Participants were weighed in their underwear, and their height was measured with a fixed stadiometer (Seca scale, Mod: 8777021094, S/N: 5877248124885, Seca Deutschland, Hamburg, Germany). A dual-energy X-ray absorptiometry (Lunar iDXA, enCORE Software, version 14.10.022; GE Healthcare, Madison, WI, USA) performing a three-site scan was used to measure body composition (fat mass, percent body fat (%BF), lean body mass, visceral adipose tissue (VAT), android-to-gynoid fat mass ratio (AG ratio), and bone mineral density (lumbar area (L2–L4); femoral neck, trochanter, and shaft (proximal femur); whole body). All data were analyzed according to previously established guidelines [35]. 

We evaluated visceral adipose tissue (VAT) according to a cut-off for healthy VAT level of ≤300 g, based on previous findings of increased risk for high levels of triglycerides and fasting insulin levels with VAT above this threshold [36]. An optimal level of body fat percentage was defined as <33% [2,37], and masked obesity was defined as having a normal body weight according to the BMI scale (i.e., <25) but with high levels of body fat percentage (i.e., ≥33%) [2].

#### 2.3.2. Cardio Respiratory Fitness (CRF)

After a breakfast meal, CRF was measured by performing a cardiopulmonary exercise test on a treadmill (ELG 90/200 Sports; Woodway, Weil am Rhein, Germany) with an incremental modified Balke protocol until exhaustion [38]. Gas exchange was measured using a breath-by-breath gas analysis system (OxyconPro analyzer; Jaeger, Würtzburg, Germany) with a Hans Rudolph two-way breathing mask (2700 series; Hans Rudolph, Kansas City, KS, USA). Measures of respiratory exchange ratio (RER) ≥ 1.10, and lactate concentration ≥ 7.0 mmol/L measured 1 min after test termination and analyzed immediately in a 1500-Sport-lactate analyzer (YSI, Yellow Springs Instruments, Yellow Springs, OH), were required to ensure a valid measure of peak oxygen uptake (VO_2_ peak) [39]. A Borg scale rating ≥17 was additionally required to approve the test result [40,41].

We evaluated CRF as low according to the suggestion from a previous publication that found an increased risk of having a cluster of risk factors for cardiovascular diseases if VO2peak ≤ 35.1 mL×kg BW×min [42].

#### 2.3.3. Maximal Muscle Strength

Maximal strength tests (one repetition maximum, 1RM) followed the cardiopulmonary exercise test in the following order: squats in Smith machine, bench press, and seated cable row. These three tests were performed according to predefined performance criteria and initiated with standardized warm-up sets of 10, 8, 6, and 4 repetitions.

#### 2.3.4. NCD Risk Profile

We categorized participants into high-risk groups for NCD if they matched at least two out of three categories for high-risk evaluation: (1) having (either/or) high BMI, high body fat percentage, or masked obesity; (2) having high VAT levels; and/or (3) having low VO2peak.

### 2.4. Ethics

Active informed consent was required, and participants needed a conformation from their general practitioner that entering the study was medically safe. The study was approved by the Norwegian Regional Committee for Medical and Health Research Ethics (ID: 2013/1871) and registered in Clinical Trials (ID: NCT02079935).

### 2.5. Statistics

All analyses were conducted in SPSS version 24 (IBM, Armonk, NY, USA). Linear mixed regression models were built to estimate the between-group differences (PED-t vs. CBT) and the within-group changes (baseline vs. any of the three post-test measures). This analysis yields relatively unbiased estimates despite drop out (i.e., in the event that data are missing completely at random or missing at random). Moreover, it can be safely used without conducting multiple imputations beforehand [43]. Standard errors were estimated with the restricted maximum likelihood function, and type III *F*-tests were preferred. Dependency in the repeated outcome measures was accounted for by including a random intercept factor. The fixed factors were: *Group* (0-PED-t, 1-CBT), representing the overall treatment difference; *Time* (repeated measures), representing change across measurements; and the *Group*×*Time* interaction, which was used in order to detect treatment differences at certain time points only. The between-group analyses used the baseline values as a covariate to increase the statistical power [44]. Differences between the treatment arms were examined with planned comparisons at each time point (least square difference tests). The within-group analyses included all four measurements in the *Time* factor. Due to the number of tests, differences with *p*-values < 0.01 were considered to be significant. A comparable statistical approach was used for the dichotomous outcome variables, replacing the analysis with a generalized linear model using a binominal distribution and logit link function (reference coded 0). Degrees of freedom were computed using Satterthwaite approximation. The outcome data are presented as estimated means including 99% confidence intervals. 

Standardized Hedge’s *g* effect sizes for continuous data were calculated as a ratio of the estimated means (extracted from the mixed model) to the observed pooled standard deviations (SDs). Values around 0.2, 0.5, and 0.8 were interpreted as weak, medium, and strong effect sizes, respectively [45].

Attrition rates were analyzed separately for each time (T2–T4) with independent *t*-test or chi-square analyses, as appropriate. A significance level of *p* < 0.05 were used for all *t*-tests or Mann-Whitney tests. 

## 3. Results

### 3.1. Attrition Analysis

Drop-outs were less physically active (lower number of counts per minute in moderate-to-vigorous intensity of physical activity, MVPA, (*p* = 0.02)) and significantly more drop-outs had low femur bone mineral density (BMD) values (*p* = 0.02). Significantly more CBT-participants were lost to follow-up both at T3 (PED-t = 19 (24.4%), CBT = 37 (47.4%), *p* = 0.005) and T4 (PED-t = 20 (25.6%), CBT = 39 (50.0%), *p* = 0.006).

### 3.2. Demographics

Demographic information is presented in Table 2. The two treatment groups did not differ in any of the demographic variables. The mean (SD) attendance rate to therapist-lead PED-t sessions was 80.6% (11.4) and 82.1% (45.7) (*p* = 0.79) in CBT. In PED-t, the adherence rate to exercise sessions (supervised in therapy + unsupervised used for homework) was 69.8% for resistance exercise and 56.7% for HIIT. 

### 3.3. Body Composition

**Figure 2:**
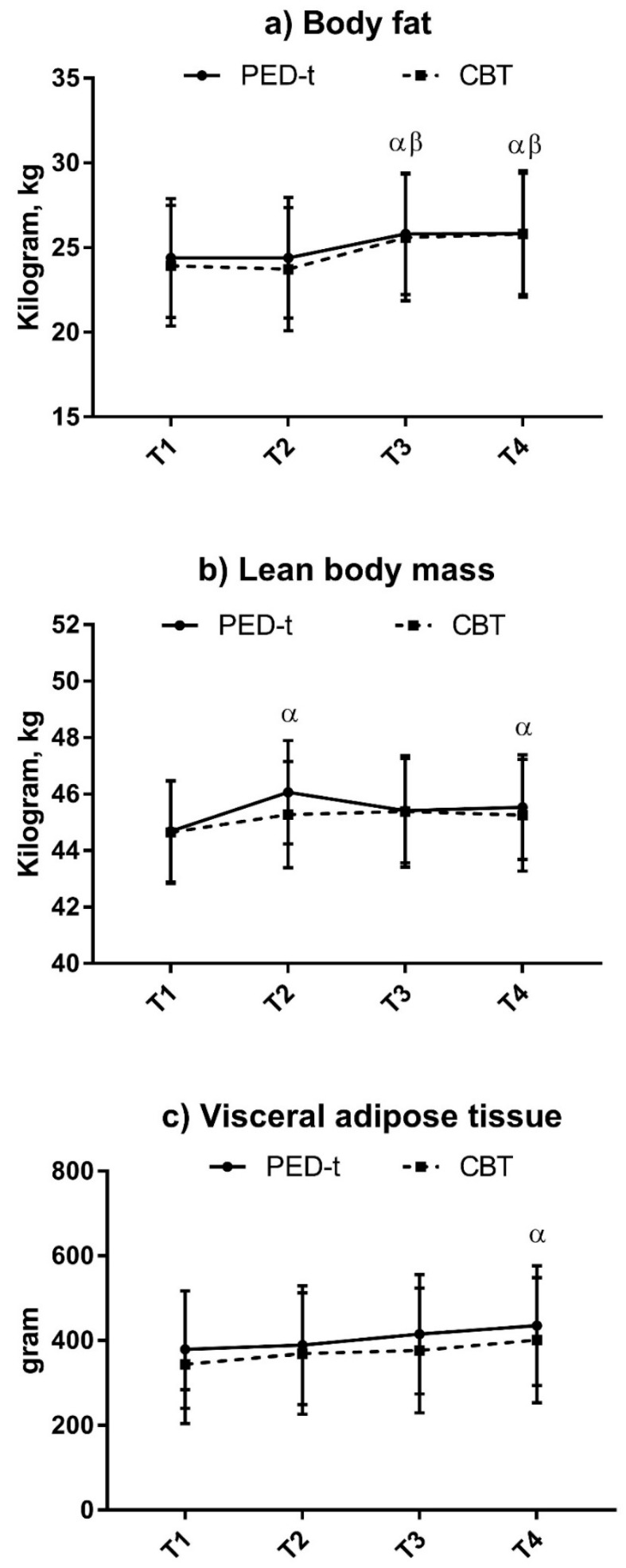
Changes in soft tissue body composition after PED-t or CBT. Results are estimated means (99% CI). (**a**) Changes in total body fat (kg); (**b**) changes in lean body mass (kg); (**c**) changes in visceral adipose tissue (gram). PED-t: Physical Exercise and Dietary therapy; CBT: Cognitive Behavior Therapy; 99% CI: 99% confidence interval; T1: baseline; T2: post-treatment; T3: 6 months post-treatment; T4: 12 months post-treatment; α: significant within-group change from T1 in PED-t (*p* < 0.01); β: significant within-group change from T1 in CBT (*p* < 0.01).

**Figure 3:**
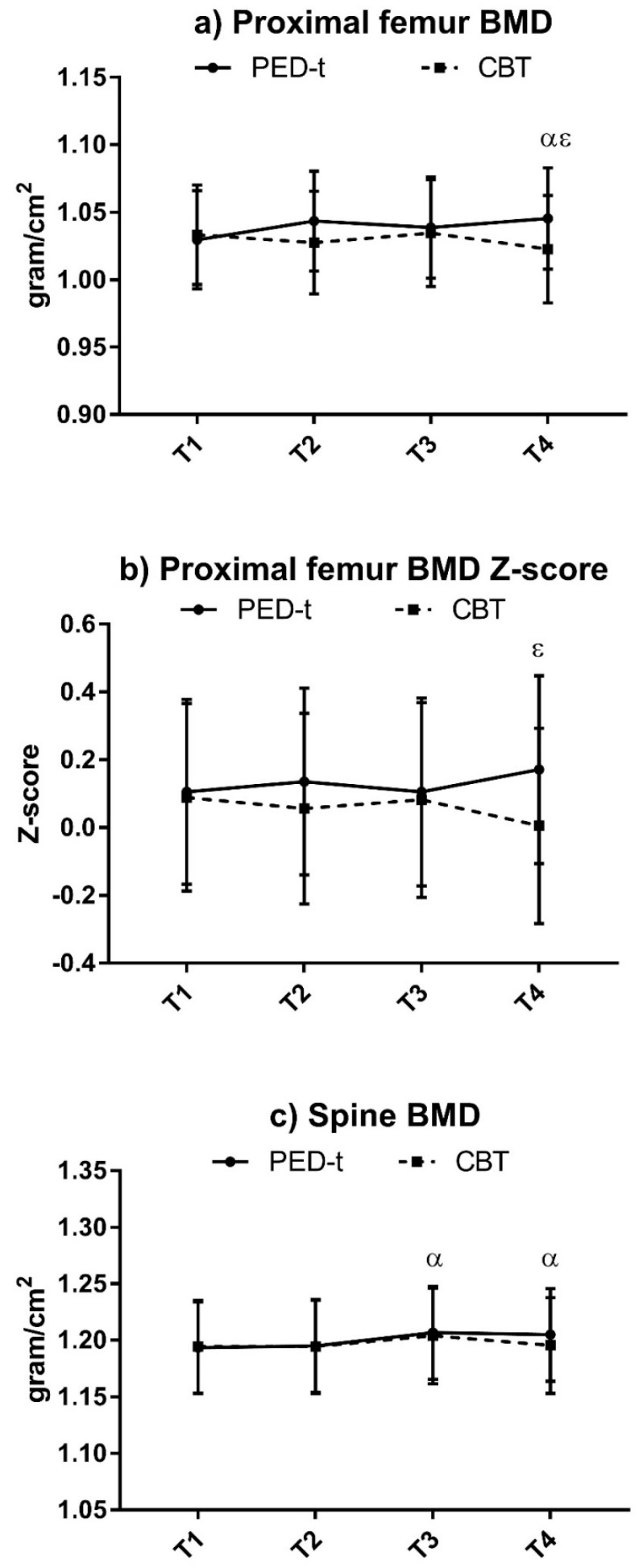
Changes in proximal femur bone mineral density (BMD), proximal femur BMD *Z*-score, and spine BMD after PED-t or CBT. Results are estimated means (99% CI). (**a**) Changes in proximal femur BMD (gram/cm^2^); (**b**) changes in proximal femur BMD *Z*-score; (**c**) changes in spine BMD (gram/cm^2^). PED-t: Physical Exercise and Dietary therapy; CBT: Cognitive Behavior Therapy; 99% CI: 99% confidence interval; BMD: bone mineral density; T1: baseline; T2: post-treatment; T3: 6 months post-treatment; T4: 12 months post-treatment; α: significant within-group change from T1 in PED-t (*p* < 0.01); ε: significant between-group difference (*p* < 0.01).

Changes in body composition are presented in Figure 2 and Figure 3. 

Mean BMI (99% CI) in PED-t increased to 25.64 (24.1, 27.1) at T2, 26.0 (24.5, 27.5) at T3, and 26.0 (24.5, 27.6) at T4. Only T3 (*g* = −0.39, *p* = 0.001) and T4 (*g* = −0.4, *p* = 0.002) were statistical different from baseline. There were corresponding findings in CBT, with levels of 25.2 (23.7, 26.8), 25.8 (24.3, 27.4), and 26.2 (24.6, 27.8), respectively, with only T4 different from baseline (*g* = −0.36, *p* = 0.004). 

The within-group change in total body fat was significant at T3 (*g* = −0.38, *p* = 0.003) and T4 (*g* = −0.31, *p* = 0.003) in PED-t, and in CBT (*g* = −0.34, *p* = 0.006, and *g* = −0.36, *p* = 0.002, respectively). The numbers with masked obesity were 6.5% (99% CI: 2.0, 1.9) in PED-t and 5.3% (99% CI: 1.4, 1.8) in CBT, with no change by time in either group.

The within-group change in lean body mass (LBM) was significant at T2 (*g* = −0.98, *p* < 0.001) and T4 (*g* = −0.47, *p* = 0.01) in PED-t, while no change was found in CBT.

The within-group change in total VAT was significant at T4 in PED-t (*g* = −0.29, *p* = 0.004), with no significant change in CBT at any time. The numbers with VAT above recommended levels at T1 were 35.1% (99% CI: 22.4, 50.3) in PED-t and 36.0% (99% CI: 23.0, 51.5) in CBT, with no change by time in either group.

There were no between-group differences at any time in soft tissue body composition.

PED-t improved mean spine BMD at T3 (*g* = −0.45, *p* = 0.004) and T4 (*g* = −0.31, *p* = 0.01), and mean proximal femur BMD at T4 (*g* = −0.37, *p* = 0.01) (Figure 3). There were no changes in mean spine or proximal femur BMD after CBT. The occurrence of low proximal femur BMD at baseline was 1.3% (99% CI: 0.0, 55.7) in PED-t and 1.3% (99% CI: 0.2, 9.0) in CBT, with no change by time (T2–T4) in either group. There were corresponding findings for low spine BMD, with 5.2% (99% CI: 1.5, 16.8) in PED-t and 6.7% (99% CI: 2.2, 18.8) in CBT at T1, respectively.

No differences between groups were found at any time, other than a better score in proximal femur BMD (*g* = 0.19, *p* = 0.01) and proximal femur BMD *Z*-score (*g* = 0.19, *p* = 0.007) in PED-t compared to CBT at T4.

### 3.4. Physical Fitness

Effects from the two therapies on physical fitness are presented in Table 3. 

#### 3.4.1. 1RM

PED-t improved 1RM in squat, bench press, and seated row at all time points, with medium-to-large effect sizes. CBT improved in 1RM squat at all time points with small effect sizes, but resulted in no other observed 1RM improvements. 

There were small-to-medium between-group differences for all strength tests at all time points, except no difference for 1RM squat at T3.

#### 3.4.2. CRF

There was a small but significant within-group increase in CRF (in absolute terms; L × min)) after PED-t, but with no further effect from the PED-t during follow-up (T3–T4) (Table 3). No change in CRF after CBT occurred, and there were no between-group differences at any time. 

### 3.5. Risk of NCDs

In the evaluation of NCD risk, two participants impaired their risk profile during the treatment period in PED-t, while five participants improved. In total, 33 participants in PED-t remained without any high-risk NCD profile during treatment, while 19 participants remained in the high-risk group. The corresponding findings in CBT were one impairment, three improvements, 28 without any risk profile during treatment, and 17 remaining in the high-risk group.

## 4. Discussion

The aim of this study was to examine the immediate and long-term effects of CBT versus a physical exercise and dietary therapy (PED-t) on physical fitness, body composition, and the risk of NCDs. We found improvements in BMD, long-term increased maximal muscle strength, and short-term increased peak oxygen uptake after a treatment period with PED-t, while CBT did not result in comparable results. The overall study findings could not support the hypothesis that the PED-t provided sufficient improvements in physical fitness that actually lowered the risk of NCDs. 

No improvement after CBT was expected. Here, healthy living was addressed, but the treatment did not consist of practical elements to help participants to improve nutrition and establish or maintain physical activity. It was unexpected, however, to observe no changes in soft BC and risk of NCDs after the PED-t; these results require some further considerations. First, it needs to be emphasized that the main aim of the PED-t was not to lower the risk of NCD, but to reduce eating disorder symptoms like irregular eating patterns and concerns about body and weight. Thus, learning how to achieve healthy living (i.e., adequate physical activity and a healthy diet composition) may be considered as positive side effects. To some extent, such side effects have been achieved through a shift of focus from overvaluation of the need to control body figure and weight towards attaining health and fitness [28]. In retrospect, however, given the low baseline values on parameters indicating an elevated risk of NCDs, it may have been overly optimistic to expect a change in physical activity and nutrition that really would lower this risk. Hence, findings that the PED-t may bring about a relaxation of dietary restrictions and compulsive physical exercise [27,28] may have counteracted any potential effect from PED-t on weight reduction and risk of NCDs. Secondly, the negative findings may, to some extent, also be explained by suboptimal treatment compliance. Thus, the participants in the PED-t, following an exercise program designed to affect BC (resistance exercise), as well as CRF and VAT (HIIT), reported unsatisfactory compliance to the program (i.e., ~70% of the RET and ~57% of the HIIT). 

The deterioration of soft tissue BC, with increases in VAT, body fat, and BMI above recommendations [2,36,46] at the follow-ups, raises concern for the health of females under and after any kind of treatment for BN and BED, and the PED-t approach included herein. Such a concern highlights that the need to improve physical fitness and BC should be focused upon in the treatment of BN and BED. In a previous PED-t publication we showed that such a focus does not initiate or maintain excessive and obsessive physical exercise frequently observed in BN and BED [27]. Hence, there is empirical evidence refuting a main clinical reluctance to incorporate guided physical activity in the treatment of eating disorders. 

An important limitation to our findings and conclusions is the skewed and high attrition rate during follow-up, with more participants being lost to follow-up in CBT compared to PED-t. The strengths of the study are the randomized design, the follow-up period, and the inclusion of participants from a real-life setting. As our findings show, maintaining lifestyle changes necessary for improvement of health is difficult in such real-life settings for persons suffering from BN or BED.

An important implication from the present study is that implementing guided physical exercise and dietary therapy is not sufficient to improve physical health in these patient groups. A recent systematic review of population-based studies on overweight has reported small but significant effects of combining CBT with a motivating interview (MI) [47]. Within the theoretical realm of self-determination theory, MI serves the purpose of internalizing treatment goals, while CBT may be effective in combating dysfunctional cognitions [48]. Such cognitions may comprise beliefs about not being able to reach treatment goals (i.e., low self-efficacy) as well as undue concerns about body, weight, and shape and the need to control eating as well as ambivalence towards change. The behavioral components of CBT serve the overall purpose of promoting self-regulation, a purpose that has been reported as being useful in the treatment of obesity [49]. However, future studies should examine if a combination of cognitive and behavioral approaches to lifestyle change improves long-term adherence to health-related exercise and eating behavior, and thus reduces risk of NCDs in persons with BN or BED to a greater extent than single-component interventions. A task for future randomized controlled studies may then be to explore the efficacy of various combinations of MI, CBT, and PED-t in the treatment of eating disorders as well as other disorders where poor physical fitness and a risk of NCDs is an issue of concern. 

## Figures and Tables

**Table 1:** Overview of the exercise module of the PED-t treatment. Resistance load is given as number of repetition maximum (RM).

		Supervised Exercise	Unsupervised Exercise	
Week	Micro Cycle	Resistance Exercise	Interval Running	Resistance Exercise
1–3	1	10RM	Pyramid interval (1–3 min intervals)	10RM
4–7	2	8RM	Pyramid interval (1–3 min intervals)	10RM
8–11	3	6RM	Pyramid interval (1–3 min intervals)	10RM
12–14	4	4RM	Pyramid interval (1–4 min intervals)	10RM
15–16	5	2RM	Pyramid interval (1–4 min intervals)	10RM

**Table 2:** Demographic description of participants in therapy groups. Results are mean (SD).

	PED-t	CBT
Age, years	28.3 (6.2)	27.8 (5.3)
BMI, kg × height^−1^	25.3 (5.1)	25.4 (4.6)
EDE-q, total score	3.7 (0.9)	3.7 (1.0)
Duration of illness, years	12.9 (7.5)	11.9 (6.7)
Bulimia nervosa, *n* (%)	51 (65.4)	50 (66.7)
Binge eating disorder, *n* (%)	27 (34.6)	25 (33.3)

**Note**: PED-t: Physical Exercise and Dietary therapy; CBT: Cognitive Behavior Therapy; BMI: Body Mass Index; EDE-q: Eating Disorder Examination questionnaire.

**Table 3:** VO_2_peak absolute, VO_2_peak relative to BW, and maximal strength (1RM) in squats, bench press, and seated row at T1–T4. Results are estimated means (99% CI), and significant within-group changes are marked with asterisks.

					Between Effects, *p*-Value, Effect Size (g)		
	T1	T2	T3	T4	T2	T3	T4
VO_2_peak _absolute_ (L × min^−1^)							
PED-t	2.67 (2.53, 2.81)	2.78 * (2.63, 2.93) *g* = 0.22	2.72 (2.57, 2.87)	2.77 (2.61, 2.92)			
CBT	2.73 (2.59, 2.88)	2.77 (2.61, 2.93)	2.78 (2.59, 2.96)	2.80 (2.62, 2.98)	*n.s.*	*n.s.*	*n.s.*
VO_2_peak _relative_ (mL × kg^−1^ × min^−1^)							
PED-t	38.25 (35.87, 40.63)	39.47 (36.92, 42.02)	38.35 (35.70, 41.00)	39.01 (36.30, 41.72)			
CBT	39.01 (36.49, 41.54)	40.35 (37.63, 43.08)	39.13 (35.95, 42.32)	39.24 (36.14, 42.33)	*n.s.*	*n.s.*	*n.s.*
Squat, 1RM (kg)							
PED-t	63.95 (58.33, 69.56)	78.60 * (72.81, 84.39) *g* = −0.83	73.89 * (67.94, 79.85) *g* = −0.56	74.98 * (68.96, 81.00) *g* = −0.71			
CBT	64.21 (58.37, 70.06)	67.15 * (61.06, 73.24) *g* = −0.15	70.07 ** (63.41, 76.72) *g* = −0.30	69.31 * (62.65, 75.97) *g* = −0.26	11.78 (7.42, 16.12) *g* = 0.65 *p* < 0.001	*n.s.*	6.10 (0.84, 11.36) *g* = 0.31 *p* = 0.003
Bench press, 1RM (kg)							
PED-t	37.02 (34.07, 39.97)	45.22 * (42.18, 48.26) *g* = −0.86	42.68 * (39.56, 45.79) *g* = −0.62	43.77 * (40.62, 46.93) *g* = −0.74			
CBT	38.32 (35.28, 41.37)	38.99 (35.80, 42.18)	39.81 (36.37, 43.26)	39.35 (35.90, 42.79)	7.28 (4.92, 9.63) *g* = 0.63 *p* < 0.001	4.12 (1.41, 6.84) *g* = 0.30 *p* < 0.001	5.60 (2.85, 8.35) *g* = 0.43 *p* < 0.001
Seated row, 1RM (kg)							
PED-t	34.38 (32.41, 36.34)	38.41 * (36.36, 40.46) *g* = −0.65	37.50 * (35.36, 39.64) *g* = −0.52	37.22 * (35.05, 39.40) *g* = −0.46			
CBT	34.56 (32.54, 36.58)	35.20 (33.06, 37.34)	35.03 (32.66, 37.41)	34.73 (32.31, 37.14)	3.39 (1.45, 5.34) *g* = 0.52 *p* < 0.001	2.58 (0.35, 4.81) *g* = 0.41 *p* = 0.003	2.74 (0.45, 5.02) *g* = 0.40 *p* = 0.002

PED-t: Physical Exercise and Dietary therapy; CBT: Cognitive Behavior Therapy; BW: Body Weight; 1RM: one repetition maximum; VO_2_peak: peak maximal oxygen uptake; 99% CI: 99% confidence interval; g: Hedges g (effect size); T1: baseline; T2: post-treatment; T3: 6 months post-treatment; T4: 12 months post-treatment; n.s., non-significant; * *p* < 0.001; ** *p* = 0.01.
